# Case Report: Radical urethrectomy with partial cystectomy and bladder outlet reconstruction in giant female urethral adenocarcinoma infiltrating the bladder: A rare case report.

**DOI:** 10.12688/f1000research.163880.1

**Published:** 2025-07-28

**Authors:** Rio Rahmadi, Utari Mudhia Arisa Putri, Hendra Herman, Wendi Rachman, Randy Fauzan, Ardiansyah Periadi Sitompul

**Affiliations:** 1 Urology Department, Raden Mattaher Hospital, Faculty of Medicine, Universitas Jambi, universitas jambi, Jambi, Indonesia, Indonesia; 2 Urology Department, Cipto Mangunkusumo Hospital/Faculty of Medicine, Universitas Indonesia, university of indonesia, Jakarta, Indonesia, Indonesia

**Keywords:** Keywords: Female urethral adenocarcinoma, radical urethrectomy, partial cystectomy, urethral cancer management

## Abstract

**Introduction:**

Female urethral adenocarcinoma (FUA) is an exceptionally rare and aggressive malignancy, accounting for less than 0.02% of all cancers in women. Its nonspecific symptoms often lead to delayed diagnosis, with many cases detected at advanced stages. The rarity of FUA, particularly when presenting with a large mass, underscores the challenges in developing standardized treatment protocols.

**Case Presentation:**

A 65-year-old woman presented with urinary retention. Clinical examination revealed a large mass obstructing the urethral orifice. A computed tomography (CT) scan showed a malignant mass involving the entire length of urethra, with no signs of metastasis. Percutaneous cystostomy was performed, and cystoscopy through the cystostomy access revealed tumor infiltration into the anterior bladder wall, approximately 2 cm from the bladder neck. A radical urethrectomy with partial cystectomy and bladder outlet reconstruction was performed via a transurethral approach, with antegrade cystoscopy guidance. The bladder outlet was reconstructed using a segment of the anterior bladder wall to facilitate voiding through the orthotopic site with a Foley catheter. Pathology confirmed urethral adenocarcinoma with clear surgical margins. Neither radiation nor chemotherapy was administered. At the 1-year follow-up, the patient reported satisfaction with her quality of life and showed no signs of recurrence or metastasis.

**Conclusion:**

This case highlights the feasibility of bladder-preserving surgical techniques in giant FUA with bladder infiltration. The approach achieved oncological control while maintaining the patient’s quality of life. Bladder outlet reconstruction provided satisfactory functional outcomes and eliminated the need for suprapubic urinary diversion.

## Introduction

Female urethral adenocarcinoma (FUA) is an exceptionally rare and aggressive malignancy, representing only 0.02% of all cancers and less than 1% of genitourinary malignancies in women. ^1^ The etiology of primary urethral carcinoma (PUC) in women is often associated with recurrent urinary tract infections and urethral diverticula. Histologically, adenocarcinomas comprise 8–10% of primary female urethral carcinomas, alongside squamous cell carcinoma (70%) and transitional cell carcinoma (20%). ^1, 2^

The diagnosis of FUA is often delayed because of its non-specific clinical presentations. Hence, it is frequently detected at an advanced stage and contributes to a higher cancer-specific mortality rate in women. ^3^ A comprehensive diagnostic approach, including urethrocystoscopy with biopsy, urinary cytology, chest and abdominal CT scans, and pelvic MRI, is essential for identifying and staging FUA. However, due to its rarity, there is still no definitive consensus on the recommended management strategies for FUA. ^4, 5^

Bladder preservation is a critical consideration in the surgical management of FUA, as it significantly enhances quality of life by avoiding complications such as incontinence or the need for urinary diversion. Traditional radical approaches often compromise bladder function, underscoring the need for innovative techniques that achieve effective tumor removal while maintaining bladder integrity. ^3, 6^

This case report aims to present our experience of surgical approach for treating FUA that prioritizes bladder preservation, thereby improving functional outcomes and quality of life. This case report has been reported in line with the CARE Checklist. ^7^

## Case presentation

**Figure 1.**
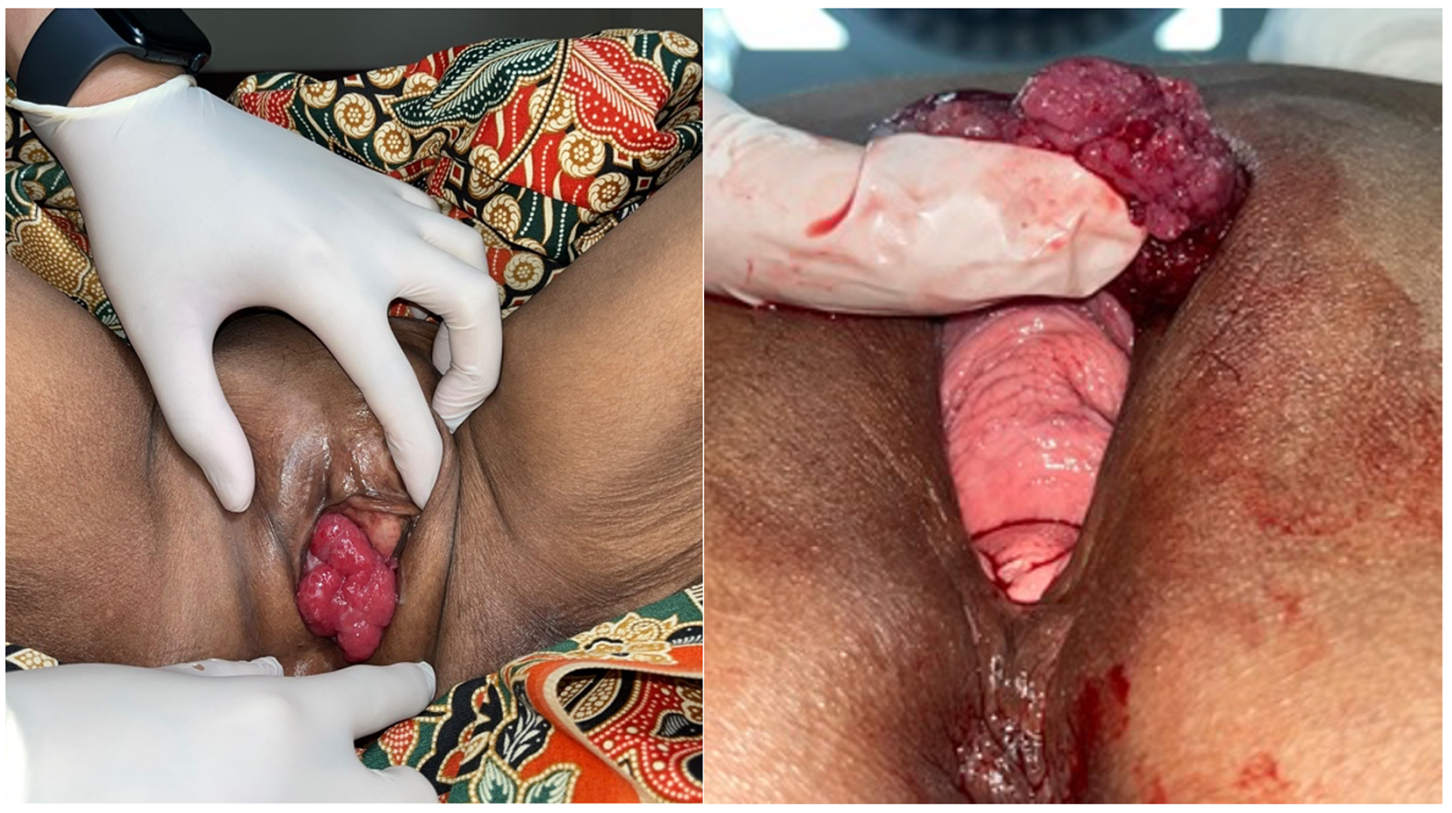
Physical examination showing a large mass obstructing the urethral orifice without involvement of the vagina.

A 65-year-old Southeast Asian woman was presented to the emergency department with urinary retention since 4 hours before admission. The patient had a history of weak stream and straining for 6 months prior. The patient denied any history of hematuria and vaginal spotting. She denied any previous surgery related to the pelvic and history of cerebrovascular disease. Her physical examination shown a full bladder and large mass covering the urethral orifice without involvement of the vagina ( Figure 1). The patient then underwent percutaneous cystostomy. A contrast-enhanced CT scan of abdomen and pelvis then performed subsequently which revealed a suspected malignant mass in the entire length of urethra, with no inguinal lymph nodes enlargement and no evidence of metastases.

**Figure 2.**
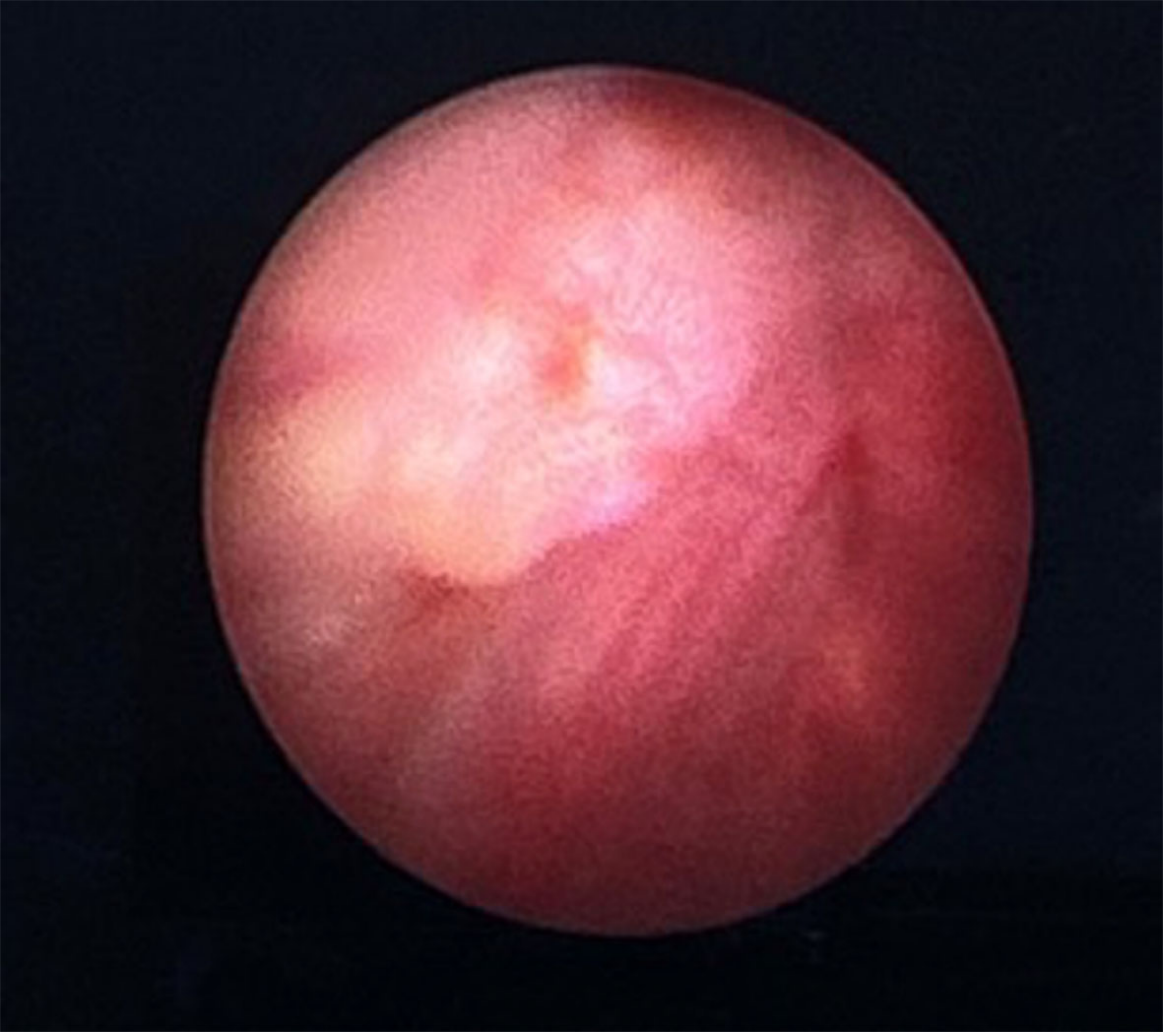
Intraoperative cystoscopy showing a tumor infiltration near the anterior portion of bladder neck.

**Figure 3.**
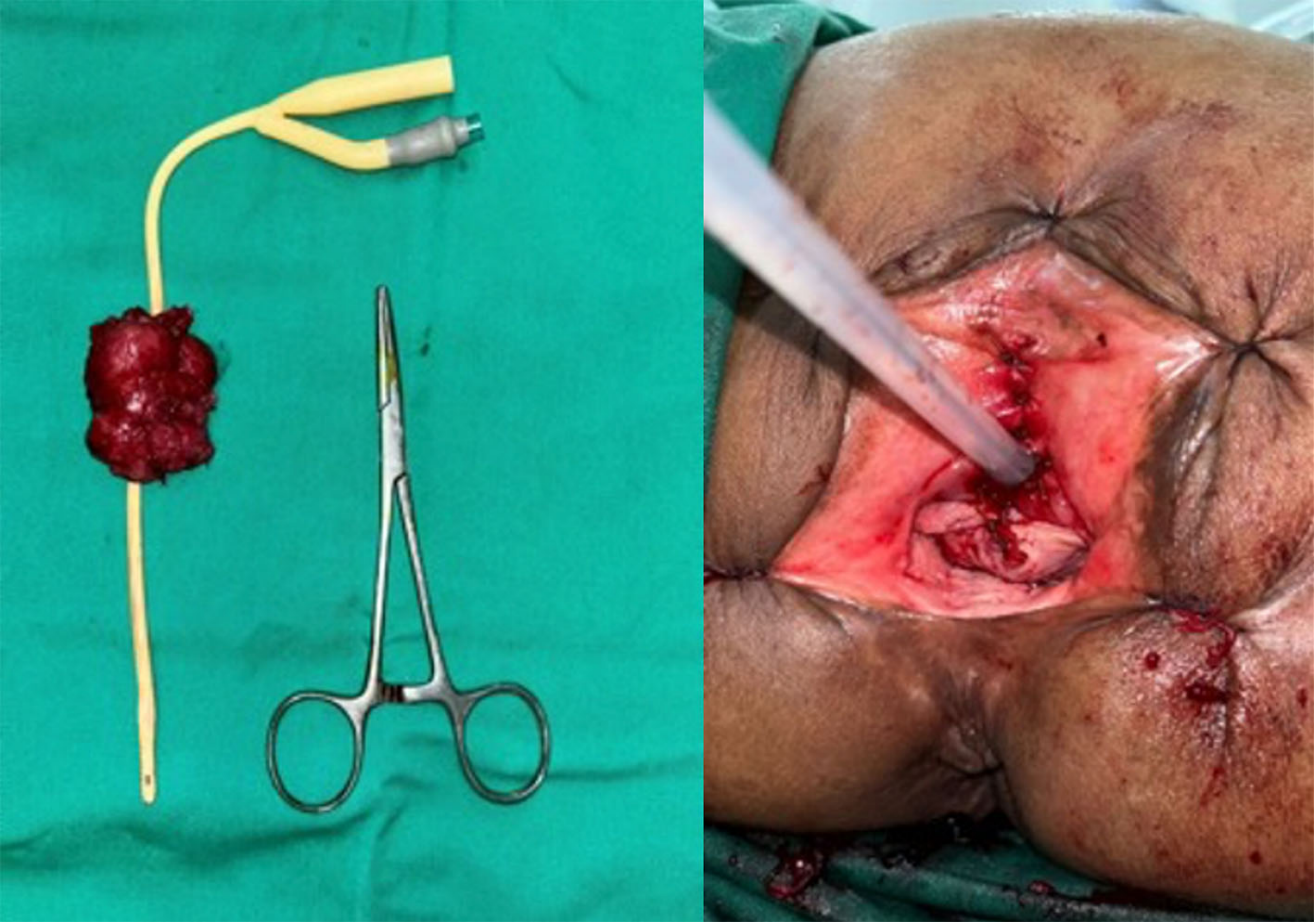
Left: Resected specimen of urethral mass and the anterior portion of bladder, Right: Post operative clinical appearance following bladder outlet reconstruction.

The patient was scheduled for a cystoscopy and urethral mass excision two days later. Cystoscope was inserted through the cystostomy access, showing a tumor infiltration to the anterior wall of the bladder approximately 2 cm from the bladder neck ( Figure 2). Radical urethrectomy and partial cystectomy were then performed through a transurethral approach with antegrade cystoscopy guidance. The excision was extended to the bladder neck and a portion of the anterior segment of the bladder wall which infiltrated by the tumor. We decided to do the bladder preservation and bladder neck reconstruction using remaining anterior bladder wall with the intention to facilitate voiding through the orthotopic site with a Foley catheter, thereby avoiding the need for a permanent suprapubic diversion. The base of the bladder was pulled through the former urethral orifice and sutured ( Figure 3). The surgery was performed without complications and the patient was discharged home within 5 days using silicone foley catheter. The pathology report revealed a urethral adenocarcinoma with clear surgical margins. Neither radiation nor chemotherapy were administered. At the 1-year follow-up, the patient reported satisfaction with her quality of life. The CT scan evaluation showed no signs of recurrence or metastasis.

## Discussion

Female urethral adenocarcinoma (FUA) is an extremely uncommon cancer, which its rarity often contributes to delayed diagnosis and suboptimal outcomes. Among the histological variants, mucinous adenocarcinoma is more frequently observed compared to clear cell adenocarcinoma. ^8^

FUA often mimics benign urinary conditions like urinary tract infections (UTIs), making diagnosis particularly challenging. Common presenting symptoms, including painful urination (dysuria), increased urinary frequency, or the presence of a palpable urethral mass, are non-specific and can easily be mistaken for less severe conditions. These diagnostic ambiguities frequently result in delays, allowing the disease to progress to advanced stages by the time it is detected. ^6, 9^

A comprehensive diagnostic strategy is critical for identifying and staging FUA. ^1^ Physical examination and initial symptoms: a detailed physical examination can uncover signs such as a urethral mass or abnormal tissue structure, typically presenting with obstructive urinary symptoms that prompt further investigation. ^2^ Role of imaging: Advanced imaging techniques like CT scans, MRI, and ultrasound play a vital role in assessing tumor size, location, and extent, as well as identifying any potential lymph node involvement or distant metastasis. ^3^ Cystoscopy and Biopsy: Cystoscopy provides direct visualization of the urethra and enables targeted biopsy, which is crucial for confirming the adenocarcinoma diagnosis histologically. This step is indispensable for determining the appropriate treatment pathway. ^10, 11^

In the published cases, treatment differs in the radicality of the surgery, from excision with or without the addition of radiation therapy to extensive surgery including cystourethrectomy, vaginectomy, vulvectomy, and lymph node dissections. ^12^ Early surgical treatment with or without adjuvant radiotherapy appears to be the best option in cases of small, organ-confined disease of urethral adenocarcinoma. Firmansyah et al. ^13^ reported a case of urethral mucinous adenocarcinoma treated with partial urethrectomy. The histology reported a negative surgical resection, however the CT evaluation showed tumor recurrence in 18 months follow up.

Traditional surgical treatment for female urethral adenocarcinoma typically involves a removal of all the periurethral tissue from the bulbocavernosus muscle bilaterally and distally, with a cylinder of all adjacent soft tissue up to the pubic symphysis and bladder neck, resulting in bladder neck closure and vesicostomy for the urinary diversion. ^14^ Meanwhile radical urethrectomy combined with bladder removal (cystectomy) is often employed in advanced cases where the cancer has spread to nearby tissues. While the primary goal of this procedure is to achieve clear surgical margins and completely remove the tumor, it is associated with significant risks and complications. ^15^

One major complication of this traditional approach is incontinence, which can severely affect a patient’s quality of life by leading to the loss of urinary control. Additionally, the removal of the bladder necessitates urinary diversion methods, such as the creation of an ileal conduit. These diversion techniques can introduce further issues, including a higher risk of urinary tract infections and metabolic disturbances. ^15, 16^

In recent years, advancements in surgical techniques have paved the way for more innovative approaches, such as transvaginal radical urethrectomy with bladder preservation. This method offers a promising alternative by focusing on tumor removal while retaining bladder function, which can result in significantly better postoperative outcomes. Preserving bladder integrity during surgery is particularly important for improving the patient’s quality of life. By maintaining the bladder, patients can retain normal urinary function, avoiding the physical and psychological challenges often associated with urinary diversion. Furthermore, this approach minimizes complications related to the management of urinary function. ^15– 17^

From a technical perspective, managing female urethral adenocarcinoma requires meticulous surgical execution. Tumor excision involves carefully removing the affected urethral mass while preserving healthy tissue to reduce the risk of recurrence. Following tumor removal, reconstructing the bladder neck becomes essential to maintaining continence. This reconstruction helps restore the anatomical structure and ensures proper urinary function, both of which are crucial for a positive surgical outcome. ^17^

Preserving continence after surgery is critical for a patient’s ability to remain independent in their daily life. Maintaining normal urinary function reduces the disruption to routine activities and social engagements. For patients who experience some level of urinary retention or difficulty voiding, intermittent self-catheterization (ISC) offers a practical and empowering solution. By allowing patients to manage their urinary needs independently, ISC minimizes reliance on caregivers and reduces the risks associated with permanent urinary diversion methods, thereby enhancing their quality of life. ^4, 11^

Bladder preservation also has significant long-term benefits for functional outcomes. Avoiding radical procedures like cystectomy helps patients retain their bladder’s natural function, enabling them to maintain normal urination patterns. Research suggests that women who undergo bladder-sparing surgeries report better functional outcomes over time compared to those who undergo more invasive approaches. ^4, 11^

In addition to functional benefits, bladder preservation reduces the likelihood of postoperative incontinence, a condition that can greatly impact quality of life. Retaining bladder integrity also contributes to improved psychological well-being, as patients experience greater control over their bodily functions and face less anxiety about potential accidents or the need for external devices. Furthermore, while the focus is often on immediate functional recovery, evidence indicates that bladder preservation may also support better long-term survival rates when combined with appropriate management strategies. ^4, 11^

In the management of female urethral adenocarcinoma (FUA), case reports highlight varied approaches tailored to the patient’s clinical condition, tumor stage, and comorbidities. Maestro et al. ^12^ reported a case of malignant melanoma of the female urethra treated with urethrectomy with bladder preservation and a catheterizable stoma (Yang-Monti Technique). Meanwhile, Pratama et al. ^8^ reported a case in Indonesia involving urethrocystouretherectomy (anterior exenteration), a radical surgical method employed for advanced disease with bladder involvement. While this approach achieved oncological control, it led to significant functional impairments. ^6^ In contrast, Chen et al. ^6^ described a conservative strategy involving tumor resection combined with bladder perfusion chemotherapy. This approach was chosen due to the patient’s advanced age and poor overall health, prioritizing quality of life over complete tumor eradication. While the method offered palliative benefits, survival outcomes were limited, illustrating the feasibility of less aggressive treatments in elderly or frail patients. ^6^ Tian et al. ^10^ explored a spectrum of strategies, emphasizing bladder preservation for localized tumors and multimodal therapy for advanced cases. In early-stage disease, localized excision enabled bladder preservation and yielded favorable outcomes with normal urinary function. For advanced cases, the authors underscored the importance of combining surgery, radiotherapy, and chemotherapy for comprehensive management. However, adherence to follow-up and adjuvant treatments proved critical, as noncompliance in one case led to recurrence and disease progression. ^10^

Bladder-preserving techniques, such as local excision or partial urethrectomy, offer better functional outcomes, including maintained continence. Tian et al. demonstrated successful results with no recurrence in a two-year follow-up for localized disease. However, these methods require consistent follow-up, as poor surveillance can lead to tumor progression and metastasis. ^10^ In contrast, traditional radical approaches like urethrectomy with bladder removal, as reported by Pratama et al., are effective for tumor clearance in advanced cases. However, they come with significant drawbacks, including the need for urinary diversion and related complications. ^5, 6, 8^

This case report highlights key limitations in managing female urethral adenocarcinoma (FUA), a rare and aggressive malignancy. The lack of standardized treatment protocols and the underexplored efficacy of multimodal therapy, combining surgery, radiotherapy, and chemotherapy, hinder consistent management. Poor adherence to follow-up protocols often leads to progression and recurrence, emphasizing the need for personalized surveillance plans. Diagnostic tools are limited, and biomarker development could improve treatment precision. While bladder-preserving techniques offer functional benefits, data on long-term quality of life and psychological impacts are scarce. ^4– 6^

## Conclusion

Due to its rarity, there are currently no established treatment protocols for treating FUA. This case highlights the feasibility of bladder-preserving surgical techniques in giant FUA with bladder infiltration. The approach achieved oncological control while maintaining the patient’s quality of life. Bladder outlet reconstruction provided satisfactory functional outcomes and eliminated the need for suprapubic urinary diversion. However, collaborative efforts and expanded studies are essential to address the complexities of this rare condition.

## Consent

Written informed consent for publication of the patient’s clinical details and clinical images was obtained from the patient. The patient has given her consent for this publication.

## Reporting guidelines

Figshare: CARE checklist for Radical Urethrectomy with Partial Cystectomy and Bladder Outlet Reconstruction in Giant Female Urethral Adenocarcinoma Infiltrating the Bladder: A Rare Case Report. https://doi.org/10.6084/m9.figshare.28732370.v1 ^7^

Data are available under the terms of the Creative Commons Zero “No rights reserved” data waiver (CC0 1.0 Public domain dedication).

## Data Availability

No data associate with this article.

## References

[1] Aleksic I Rais-Bahrami S Daugherty M : Primary urethral carcinoma: A Surveillance, Epidemiology, and End Results data analysis identifying predictors of cancer-specific survival. *Urol. Ann.* 2018 Apr-Jun;10(2):170–174. 10.4103/UA.UA_136_17 29719329 PMC5907326

[2] Derksen JW Visser O Rivière GB de la : Primary urethral carcinoma in females: an epidemiologic study on demographical factors, histological types, tumour stage and survival. *World. J. Urol.* 2013 Feb;31(1):147–153. 10.1007/s00345-012-0882-5 22614443

[3] European Association of Urology: *EAU guidelines on primary urethral carcinoma.* Arnhem, Netherlands: EAU Office, EAU;2023 [updated Mar 2023; cited 2023 Apr].

[4] Kurniawan J Seputra KP Daryanto B : Primary urethral carcinoma in female: An extremely rare case series at a single tertiary referral hospital and literature review. *Int. J. Surg. Case Rep.* 2024 Aug;121:109993. 10.1016/j.ijscr.2024.109993 38972106 PMC11277758

[5] Chen H Zou LL Dong CJ : Advanced primary urethral cancer: a case report. *J. Med. Case Rep.* 2019 Nov 29;13(1):365. 10.1186/s13256-019-2253-y 31779706 PMC6883513

[6] Dell’Atti L Galosi AB : Female Urethra Adenocarcinoma. *Clin. Genitourin. Cancer.* 2018 Apr;16(2):e263–e267. 10.1016/j.clgc.2017.10.006 29113768

[7] Mudhia U : CARE Checklist_Radical Urethrectomy with Partial Cystectomy and Bladder Outlet Reconstruction in Giant Female Urethral Adenocarcinoma Infiltrating the Bladder- A Rare Case Report.pdf. *figshare.* 2025 [cited 2025 Jun 7]. Reference Source 10.12688/f1000research.163880.2 PMC12848345 41614087

[8] Pratama ME Ismy J Kamarlis R : Female primary urethral carcinoma: A rare case report. *Int. J. Surg. Case Rep.* 2021 Aug;85:106100. 10.1016/j.ijscr.2021.106100 34311342 PMC8326724

[9] Wang X Bai P Su H : Management of primary adenocarcinoma of the female urethra: Report of two cases and review of the literature. *Oncol. Lett.* 2012;4:951–954. 10.3892/ol.2012.886 23162629 PMC3499605

[10] Tian J Zhu T Xu Z : Management of Primary Female Urethral Adenocarcinoma: Two Rare Case Reports and Literature Review. *Medicina.* 2023;59(1):109. 10.3390/medicina59010109 36676733 PMC9865078

[11] Carlock HR Spiess PE : Review on urethral cancer: what do you need to know. *AME Med. J.* 2020;5:7. 10.21037/amj.2020.01.06

[12] Maestro MA Martinez-Piñeiro L Gonzalez ER : Radical urethrectomy with bladder preservation and continent catheterizable stoma (Yang-Monti tecnique). *Indian J. Urol.* 2012 Jan;28(1):107–110. 10.4103/0970-1591.94971 22557732 PMC3339777

[13] Firmansyah MR Bramono IA Santoso RB : Case Report: A rare case of mucinous adenocarcinoma of the female urethra [version 1; peer review: 1 approved with reservations, 1 not approved]. *F1000 Res.* 2023;12:664. 10.12688/f1000research.132303.1

[14] Blaivas JG : Periurethral masses: etiology and diagnosis in a large series of women. *Obstet. Gynecol.* 2004;103:842–847. 10.1097/01.AOG.0000124848.63750.e6 15121554

[15] Hirobe M Tanaka T Shindo T : Complications within 90 days after radical cystectomy for bladder cancer: results of a multicenter prospective study in Japan. *Int. J. Clin. Oncol.* 2018;23:734–741. 10.1007/s10147-018-1245-z 29442282

[16] Laukhtina E Boehm A Peyronnet B : Urethrectomy at the time of radical cystectomy for non-metastatic urothelial carcinoma of the bladder: a collaborative multicenter study. *World. J. Urol.* 2022 Jul;40(7):1689–1696. 10.1007/s00345-022-04025-z 35596017 PMC9236994

[17] Tan WS Lamb BW Kelly JD : Complications of Radical Cystectomy and Orthotopic Reconstruction. *Adv. Urol.* 2015;2015:1–7. 10.1155/2015/323157 PMC4677163 26697063

