# Supplementary material for: Case Report: Radical urethrectomy with partial cystectomy and bladder outlet reconstruction in giant female urethral adenocarcinoma infiltrating the bladder: A rare case report
Source: F1000Res. 2026 Jan 2;14:741. Originally published 2025 Jul 28. [Version 2] doi: 10.12688/f1000research.163880.2 (PMC12848345; doi:10.12688/f1000research.163880.2)
Supplement: Peer review history

# Reviewer report: Reviewer response for version 2

**DOI:** 10.5256/f1000research.190893.r446925

**Published:** 2026-01-27

**Authors:** Andika Afriansyah

**Affiliations:** 1 RS Pusat Persahabatan, Jakarta, Indonesia

I have reviewed the revised manuscript. The authors have adequately addressed my previous concerns, and the manuscript is much improved.

Are enough details provided of any physical examination and diagnostic tests, treatment given and outcomes?

Partly

Is the case presented with sufficient detail to be useful for other practitioners?

Yes

Is sufficient discussion included of the importance of the findings and their relevance to future understanding of disease processes, diagnosis or treatment?

Partly

Is the background of the case’s history and progression described in sufficient detail?

Yes

Reviewer Expertise:

NA

I confirm that I have read this submission and believe that I have an appropriate level of expertise to confirm that it is of an acceptable scientific standard.

# Reviewer report: Reviewer response for version 2

**DOI:** 10.5256/f1000research.190893.r446924

**Published:** 2026-01-12

**Authors:** Ting Zhao

**Affiliations:** 1 Harvard Medical School, Boston, Massachusetts, USA

Author has addressed my comments.

Are enough details provided of any physical examination and diagnostic tests, treatment given and outcomes?

Yes

Is the case presented with sufficient detail to be useful for other practitioners?

Yes

Is sufficient discussion included of the importance of the findings and their relevance to future understanding of disease processes, diagnosis or treatment?

Yes

Is the background of the case’s history and progression described in sufficient detail?

Yes

Reviewer Expertise:

Urological pathology

I confirm that I have read this submission and believe that I have an appropriate level of expertise to confirm that it is of an acceptable scientific standard.

# Reviewer report: Reviewer response for version 1

**DOI:** 10.5256/f1000research.180299.r401223

**Published:** 2025-08-27

**Authors:** Andika Afriansyah

**Affiliations:** 1 RS Pusat Persahabatan, Jakarta, Indonesia

Are enough details provided of any physical examination and diagnostic tests, treatment given and outcomes?

 PARTLY

 Major comments :

 1. It appears that no preoperative biopsy was performed and the authors proceeded directly with urethrectomy. Could the authors clarify the rationale behind this decision, and whether specific clinical or technical considerations influenced this approach?

 2. Please provide the CT scan pre operatively in the image. 

 3. Please provide the TNM staging based on the CT scan.

 4. The surgical technique is innovative but insufficiently detailed—suture method, catheterization duration, and measures to avoid stenosis should be provided. Do you the anterior flap and suture it directly into the posterior urethra? Please highlight  what surgical procedure that make patient had a good continent status without any bladder neck stenosis. 

 5. Do you do the frozen  section in intraoperative? If you do the frozen section, please clarify the result

 6. The manuscript would benefit from more detailed reporting of the functional outcomes following bladder neck reconstruction. Specifically, could the authors provide information on:

- Continence status – did the patient experience stress, urge, or continuous incontinence after surgery?
- Uroflowmetry results – were objective assessments such as maximum flow rate (Qmax) or voided volume performed during follow-up?
- Post-void residual urine (PVR) – was ultrasonography or catheterization used to assess residual urine, and what were the findings?

Since one of the key novelties of this case lies in bladder preservation with outlet reconstruction, reporting these parameters would significantly strengthen the case report and its value for other practitioners. Functional outcomes are as important as oncological control in such cases, and more detail here would enhance the completeness of the report

 Minor comments :

 1. Regarding Figure 1, the current clinical photograph appears suboptimal for formal publication, as the background includes elements of the patient’s clothing that may be distracting. For clarity and professionalism, it would be advisable to replace this with a standardized clinical image, ideally using a neutral background and appropriate anonymization

 Is sufficient discussion included of the importance of the findings and their relevance to future understanding of disease processes, diagnosis or treatment?

 Partly

 Major comments

 1. Why CT scan chosen in this case not MRI? Please discuss it on the discussion part

 2. Given the rarity but aggressive nature of female urethral adenocarcinoma, evidence supports the consideration of adjuvant treatment for locally-advanced or high-risk features. For instance, several studies document that a cisplatin-based regimen (e.g., 5‑FU/cisplatin) concurrent with radiotherapy offers improved local control and disease-free survival in advanced urethral carcinoma. In this case, no chemotherapy or radiotherapy was administered postoperatively. Could the authors elaborate on the rationale behind this decision? Given the aggressive nature of female urethral adenocarcinoma and the evidence supporting multimodal therapy in selected cases, clarification regarding tumor stage, margin status, patient factors, or institutional considerations that influenced this management choice would be highly valuable.

 3. The authors’ method (using anterior bladder wall to fashion an outlet and pulling the bladder base to the orthotopic meatus) conceptually aligns with this flap‑based continence‑preserving strategy. To prove success, they should report continence measures and uroflow/PVR. Is there any stress incontinence? Please detail the suture technique (layers, material), catheterization protocol/duration, and any anti‑stenosis measures at the reconstructed bladder neck. Did you add bladder‑neck support (suspension, posterior anchoring) to protect continence?

 4. Based on your author clinical judgment, what anatomical/functional criteria (e.g., bladder capacity > 200 mL, absence of active inflammation, adequate anterior wall tissue) would you use to select patients for this technique? Why the authors choose to reconstruct the bladder neck compared to bladder neck closure and catheterizable stoma? Any pre operative judgment using functional status?

Are enough details provided of any physical examination and diagnostic tests, treatment given and outcomes?

Partly

Is the case presented with sufficient detail to be useful for other practitioners?

Yes

Is sufficient discussion included of the importance of the findings and their relevance to future understanding of disease processes, diagnosis or treatment?

Partly

Is the background of the case’s history and progression described in sufficient detail?

Yes

Reviewer Expertise:

onclogy, urooncology, functional urology, neurourology, female urology

I confirm that I have read this submission and believe that I have an appropriate level of expertise to confirm that it is of an acceptable scientific standard, however I have significant reservations, as outlined above.

# Response

**Published:** 2025-11-04

**Authors:** Utari Mudhia Arisa Putri

**Affiliations:** Urology, Not Applicable, Jakarta Barat, DKI Jakarta, Indonesia

Author Response to Reviewer 2:

 Thank you for your appreciation and comments.

 For major comments:

 1. Due to highly suspicious lesions of malignancy based on visual, endoscopic, and imaging appearance, we didn’t perform preoperative biopsy and proceeded to definitive surgery. We have added this information in the latest manuscript

 2. In the updated version, we added the CT scan pre operatively in the image and describe the TNM staging based on the CT Scan

 3. For the detailed surgical procedure, we added "The tumor-free bladder punctum was pulled through the former urethral orifice and the bladder muscle was sutured to the urogenital diaphragm using 3-0 Vicryl, interrupted in eight directions (Figure 4). The bladder mucosa is sutured to the vulvar mucosa, similar to a vesicostoma with 5-0 Chromic, interrupted in eight directions. After the suturing was done, we re-evaluate the bladder with cystoscopy to make sure the ureteral orifices remain intravesical" in the revised manucript 

 4. We do not have frozen section facilities in our hospital. To avoid a positive margin, with did the procedure with antegrade cystoscopy guidance. We have added this information in the latest manuscript

 5. In the revised version, we also explain more about the continence status. We added "At 1 month follow-up, the patient was in continuous incontinence status. This condition is expected since the sphincter was also resected during the surgery as the tumor already infiltrated the anterior bladder. But with the use of silicone catheter, we can avoid any leak and patient still can void timely through regularly clamp catheter. We didn’t performed uroflowmetry and PVR evaluation since the patient is incontinent without foley catheter. We aimed to maintain the catheter for long term usage to increase the patient quality of life.  At the 1-year follow-up, the patient reported satisfaction with the outcome of the surgery and her quality of life"

 Minor comments :

 1. Regarding Figure 1, we have updated the picture according to the standard requirements for publication.

 Major comments

 1. In this case, we decided to perform CT scan instead of MRI to assess lymph nodes and metastases better as the patient was suspected with invasive disease. We have added this information in the latest manuscript

 2. Neither radiation nor chemotherapy were administered since the patient has negative node and no distant metastasis. We added the rationale with "Due to its rarity and lack of long-term follow-up in most reports, there is no standard chemotherapy regimen validated for female urethral adenocarcinoma, especially the enteric subtype. ^19^ Radiotherapy is considered less effective for adenocarcinoma compared to squamous/urothelial cancers, yet may be used for unresectable disease, palliation, or multimodal therapy. As reported by Ge R, et al. 2024, in their 35-case series of enteric/mucinous UA, surgery was the mainstay. Radiotherapy was rarely used and not associated with durable control, suggesting limited radiosensitivity. ^2^ Quality of life may be worsen due to side effect of the chemoradiation therapy. Also, the patient showed no sign of lymph node involvement or distant metastasis, so we didn’t perform the multimodal therapy."

 3. The surgery was performed without complications and the patient was discharged home within 5 days using silicone foley catheter. The silicone catheter is left in place for 1 month to assure good healing and avoid stenosis. At 1 month follow-up, the patient was in continuous incontinence status. This condition is expected since the sphincter was also resected during the surgery as the tumor already infiltrated the anterior bladder. But with the use of silicone catheter, we can avoid any leak and patient still can void timely through regularly clamp catheter. We didn’t performed uroflowmetry and PVR evaluation since the patient is incontinent without foley catheter. We aimed to maintain the catheter for long term usage to increase the patient quality of life

 4. In this case, we avoided abdominal catheterizable stoma such as mitrofanoff considering the patient have low economic status and live in remote area, so she didn’t have access to regular CIC and adequate health care facility. It will also change the patient body anatomical that will impaired her quality of life and probably cost higher to do the routine care. Therefore, based on our clinical judgment, there are several criteria that are suitable for this approach, namely (1) Female, (2) Normal bladder capacity, (3) Infiltration of tumor is localized in the infratrigonum, (4) Good performance status/karnofsky, (5) Limited access to primary health care and CIC. We have added this information in the latest manuscript

 We hope that this revised version of paper can address every comment and opinion that can improve the quality of this manuscript.

 Thank you

# Reviewer report: Reviewer response for version 1

**DOI:** 10.5256/f1000research.180299.r401232

**Published:** 2025-08-11

**Authors:** Ting Zhao

**Affiliations:** 1 Harvard Medical School, Boston, Massachusetts, USA

Authors reported a rare case of a giant female urethral adenocarcinoma infiltrating the bladder and described their surgical approach that prioritized bladder preservation. A few comments and suggestions are provided below:

1. Please include pathology images and specify the tumor size, histologic subtype (mucinous, enteric, signet-ring cell, or NOS), and pathological stage of the urethral adenocarcinoma.
2. Consider adding a brief discussion of the pathological and molecular features of urethral adenocarcinoma, with references such as Zhao T, et al 2022 [Ref 1]; Ge R, et al 2024 [Ref 2]; and Zhao T, et al 2025 [Ref 3].
3. The term “female urethral adenocarcinoma (FUA)” is spelled out in the Introduction; therefore, it does not need to be spelled out again in the Discussion.

Are enough details provided of any physical examination and diagnostic tests, treatment given and outcomes?

Yes

Is the case presented with sufficient detail to be useful for other practitioners?

Yes

Is sufficient discussion included of the importance of the findings and their relevance to future understanding of disease processes, diagnosis or treatment?

Yes

Is the background of the case’s history and progression described in sufficient detail?

Yes

Reviewer Expertise:

Urological pathology

I confirm that I have read this submission and believe that I have an appropriate level of expertise to confirm that it is of an acceptable scientific standard, however I have significant reservations, as outlined above.

## References

[1] : Mucinous adenocarcinoma of the prostatic urethra after brachytherapy for prostatic adenocarcinoma: a case series. *Human Pathology* .2022;128: 10.1016/j.humpath.2022.07.018 101-109 10.1016/j.humpath.2022.07.018 35926810

[2] : Primary mucinous adenocarcinoma of the urethra: A clinicopathological analysis of 35 cases. *Histopathology* .2024;84(5) : 10.1111/his.15118 753-764 10.1111/his.15118 38114291

[3] : Primary urethral adenocarcinoma harbors recurrent KRAS and EGFR alterations. *Human Pathology* .2025;157: 10.1016/j.humpath.2025.105771 10.1016/j.humpath.2025.105771 40194726

# Response

**Published:** 2025-11-04

**Authors:** Utari Mudhia Arisa Putri

**Affiliations:** Urology, Not Applicable, Jakarta Barat, DKI Jakarta, Indonesia

Author Response to Reviewer 1:

 Thank you for your appreciation and comments

 1. We have updated the article with the pathology images. We also specified the tumor size, histologic subtype and pathological stage in the revised article. We add "The pathology report revealed 5x4 cm sized pT4 urethral adenocarcinoma with bladder infiltration, clear surgical margins, and enteric type histologic subtype"

 2. We added a brief discussion of the pathological of enteric subtype urethral adenocarcinoma, with Zhao T, et al 2022 as reference, in the discussion part. It is stated "Enteric/mucinous (intestinal type) subtype often show abundant extracellular mucinous and clusters of neoplastic cells floating in mucin pools. It resembles colorectal adenocarcinoma histologically and immunohistochemically but can be distinguished by lack of nuclear β-catenin and clinical context. ^"^

 3. We removed the spelled-out term “female urethral adenocarcinoma” 

 We hope that this revised version of paper can address every comment and opinion that can improve the quality of this manuscript.

 Thank you
